# Terrible triad injury of the elbow: a spectrum of theories

**DOI:** 10.1016/j.jseint.2023.03.018

**Published:** 2023-04-15

**Authors:** Pierre Laumonerie, Pierre Mansat

**Affiliations:** a Department of Orthopedics and Traumatology, Hôpital Pellegrin, Bordeaux, France; b Department of Orthopedic Surgery, Hôpital Pierre-Paul Riquet, Toulouse, France

**Keywords:** Biomechanics, Coronoid, Elbow instability, History, Radial head, Terrible triad

## Abstract

For more than one century, understanding the injury mechanism leading to the terrible triad of the elbow (TTE) was a significant challenge for surgeons. We aimed to summarize: (1) the history of the treatment of TTE and (2) the increasing scientific knowledge that supported its evolution. Five electronic databases were searched between 1920 and 2022. Results were reported as a comprehensive review of the relevant literature. Between 1940 and 1980, surgical exploration allowed observation of complex elbow instability involving both radial head, coronoid process, and ligament(s) injuries. In 1966, Osborne introduced the concept of posterolateral rotatory instability as the first mechanism injury to explain the complex elbow instability. From 1980 to 1995, a biomechanical revolution by American pioneers critically improved our understanding of elbow instability. After 1992, a few unifying theories and surgical protocols were provided, but those have divided the surgeons’ population. The formalization of the TTE treatment allowed avoiding of terrible short-term outcomes. However, post-traumatic osteoarthritis (PTOA) at long-term follow-up is still an issue. No consensual surgical protocol for the treatment of TTE has been widely accepted. While the outcomes of the TTE have been improved, the rate of PTOA at long-term follow-up is still high regardless of the treatments. The terrible triad has given way to the subtle triad with persistent microinstability of the elbow. The next challenge for elbow surgeons is to diagnose and fix this persistent subclinical instability after surgery in order to prevent the onset of PTOA.

Based on his clinical experience, Hotchkiss^10^ first defined in 1996 an injury pattern involving an elbow dislocation associated with a radial head (RH) fracture and a coronoid process (CP) fracture. He named it “The Terrible Triad injury of the elbow” because of the poor prognosis, including mainly stiffness and recurrent instability (Fig. 1).

**Figure 1:**
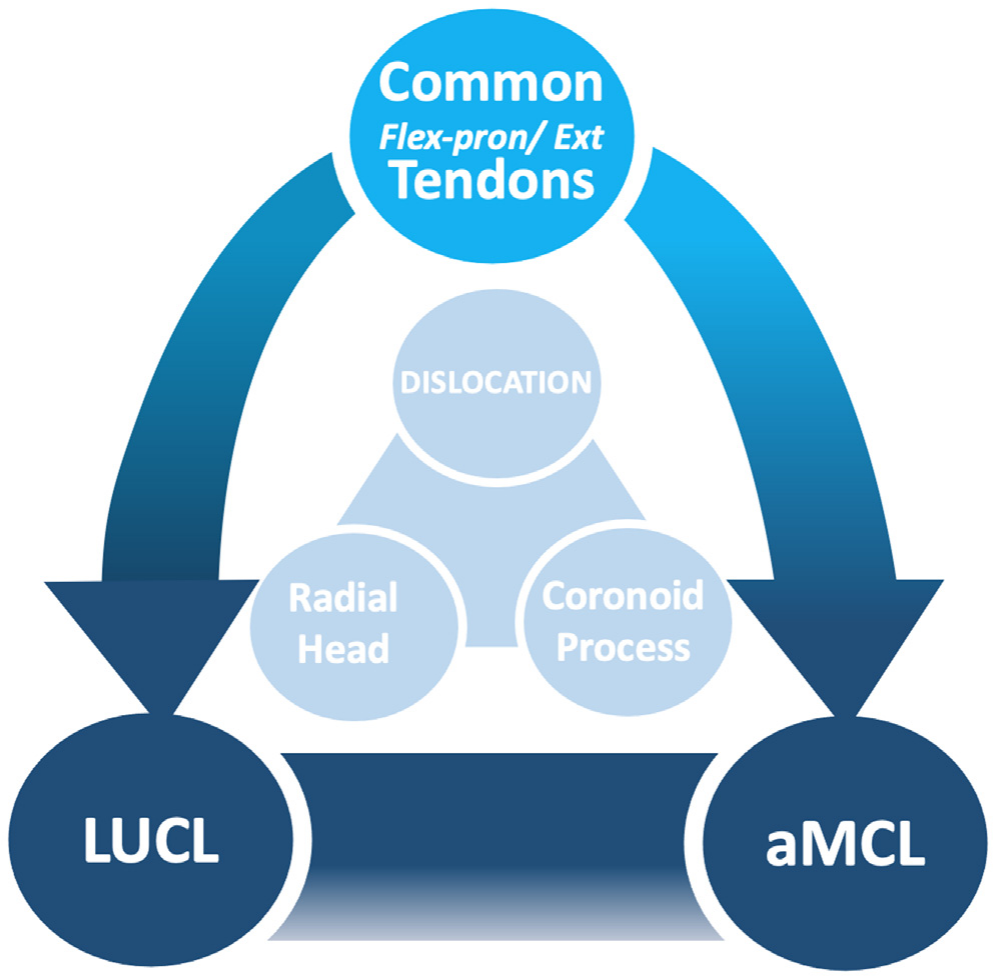
Terrible triad injury of the elbow: an injury pattern. Since 1996, the TTE of the elbow has been defined by RH and CP fractures associated with elbow dislocation (light blue circle). The growing knowledge in the biomechanics of elbow stability showed the involvement of the LCUL and aMCL, and/or the common flexor-pronator and extensor tendons originate in TTE (blue circle). *TTE*, terrible triad of the elbow; *LCUL*, lateral collateral ulnar ligament; *RH*, radial head; *CP*, coronoid process; *aMCL*, anterior medial collateral ligament.

Several theories on the terrible triad of the elbow (TTE) were then published by other surgeons after better understanding of the biomechanical condition of the elbow joint stability. Based on these theories, they have then proposed surgical protocol guidelines.^21^ However, there was no widely accepted approach for the treatment of TTE.

We aimed to summarize: (1) the history of the treatment of TTE and (2) the increasing scientific knowledge that supported its evolution.

## Methods

An electronic literature search was carried out using PubMed, Scopus, Medline, EMBASE, and the Medical Subject Headings. The search was limited to English language literature. The terms « AND » and « OR »; « arthroplasty »; «elbow dislocation»; «prosthesis »; and « radial head » were used in various combinations with “AND” and “OR” to assist in the review. The reference list of each article was also searched in order to identify additional articles pertinent to our research criteria. References from the existing literature were also queried because of the limited historical time frame inherent in these search engines. Results were reported as a comprehensive review of the relevant literature from 1920 to 2022.

## Results

### Surgical exploration (1940-1980)

In the early 20th century, closed reduction with or without RH resection in cases of unreconstructible fractures was the main treatment of TTE.^2^

According to the most popular theories, ligament laxity and/or incongruity between the trochlear notch (ie, depth) and coronoid (ie, height) were the 2 main causes of persistent elbow instability after dislocation.^23^ The goal of the initial surgical treatment was to prevent the ulna from disengaging with the trochlea by increasing the stability of the CP (eg, transfer biceps tendon to the CP, CP bone block augmentation, and direct anterior capsular repair).^23^ These surgical procedures, derived from the Bankart repair for shoulder instability, led to an incidence of 38%-63% of post-traumatic osteoarthritis (PTOA).^14^

Heterotopic ossifications of the elbow and/or wrist pain were also frequently observed after elbow dislocation regardless of these treatments. In order to prevent these complications, Speed^28^ proposed in 1941 the first RH implant consisting of a Vitallium cap placed over the radial neck. Ten years later, Essex-Lopresti et al^4^ described a series of cases of forearm instability after RH resection. The same year, Essex-Lopresti as well as Carr and Howar^1^ showed that RH replacement by an implant was required in case of RH fracture and radio-ulnar joint injury to maintain elbow stability and prevent painful wrist instability.

The medial collateral ligament (MCL), along with its anterior, posterior, and transverse bands was first described by Gutierrez et al^6^ in 1964. In this article, the authors showed that the anterior medial collateral ligament (aMCL) limited the angular opening of the humero-ulnar joint. They, therefore, speculated that the aMCL could be the primary contributor to the elbow stability in valgus.

In 1966, an injury pattern involving elbow dislocation, lateral collateral ligament complex (LCLC) injury, and fracture of the RH was described.^23^ Based on a literature review, Osborne and Cotterill provided the first unifying theory to explain simple and complex elbow dislocations. They speculated that the force of the fall on an out-stretched hand and incompletely extended elbow might induce an impaction of the coronoid against the trochlea and potentially a fracture of the CP. This vertical thrust was also converted into lateral rotation and valgus strains of the ulna by the laterally sloping surface of the medial part of the trochlea. Then, a posterior-lateral disengagement of the CP and the RH could be, therefore, observed by the induced posterolateral (PL) rotation movement of the ulna. The dislocation of the RH behind the capitulum induced stripping of the LCLC and a tear of the PL capsule. Osborne et Cotterill^23^ and then Hassman et al^7^ in 1975 showed that the postero-lateral rotatory instability (PLRI) of the elbow after TTE could be treated by repairing the LCLC. Similarly, repair of the aMCL was recommended only in cases of persistent valgus instability after repair of LCLC.^23^

### A biomechanical revolution from America (1980-1995)

The increase of biomechanical data discovered from 1980 to 1995 has critically improved our understanding of elbow instability.

In 1981, Tullos et al^29^ set out the 3 primary static constraints of the elbow to valgus stress: aMCL, RH, and CP. In 1989, Morrey and Regan^25^ developed a new classification describing the extent to which the height of the coronoid contributed to elbow stability. The classification was based on 3 fracture sizes of the CP: tip avulsion (type I), <50% (type II), and >50% (type III). For type-III CP fracture, the authors showed an increased risk of recurrent elbow dislocation and always recommended reducing and fixing it regardless of the associated ligamentous and bony injuries.

In 1987, Josefsson et al^15^ published a series of 31 simple elbow dislocations (n = 31) with 100% MCL rupture. Between 1987 and 1991, Hotchkiss^11^ and Morrey et al^20^ published additional data suggesting that the aMCL was the most critical stabilizer of the elbow throughout the flexion-extension arc. Morrey et al^19^ also showed that its contribution to elbow stability increased in cases where the RH was resected. Conversely, in the case of competent MCL, the comminuted RH could be excised without a significant increased risk of altered elbow biomechanics.^15^ Therefore, the authors supported that a RH arthroplasty was indicated to prevent gross valgus instability in case of TTE involving both unreconstructible RH fractures with aMCL deficiency. Based on this new biomechanical knowledge, the authors reclassified the RH as a secondary constraint and considered the LCLC, the aMCL, and the humero-ulnar joint as primary stabilizers of the elbow under valgus strain. However, all biomechanical studies analyzed the stability of the elbow under combined rotatory strains and motions, which did not allow us to identify the contributor to the PLRI.

In 1992, O'Driscoll et al^21^ showed that a rupture of the lateral collateral ulnar ligament (LCUL) could induce an elbow dislocation regardless of the status of the aMCL. They^21^ provided biomechanical results confirming the mechanism speculated by Osborne and Cotterill.^23^ The authors described a transient rotatory subluxation of the ulno-humeral joint after a rupture of the LCUL under a combination of an axial load, a valgus (15°) stress, a moderate internal rotation or a hyper-supination (40°) of the forearm, and a slight flexion of the elbow.^21^

### Unifying theories and spliting surgeons; population (1992-2020)

Based on his clinical experience, Hotchkiss^10^ first defined in 1996 the TTE as an injury pattern involving an elbow dislocation associated to a RH fracture and a coronoid CP fracture. The term “terrible” referred to poor treatment outcomes, including stiffness, recurrent instability, and critical arthritis (Fig. 1).

#### Spectrum of unifying theories

As a result of the inability to explain pathoanatomic features of the TTE, some authors speculated about sequential injury to unify clinical findings and posterolateral rotatory instability mechanism observed after biomechanical studies.

In 1992, O'Driscoll et al^21^ described the Horii circle illustrating the sequential soft tissue and bony injuries according to a spectrum of elbow instability. This circle starts from the lateral structures of the elbow and progresses according to 3 stages to the medial structures. Stage 1 corresponded to the PLRI caused by the rupture of the LCUL. Stage 2 corresponded to the perched elbow related to the rupture of the LCL complex associated with the injury of the elbow capsule (anterior and/or posterior). Stage 3 concerns the dislocated elbow caused by the disruption of the previous structures and the posterior (a) and then anterior (b) band of the MCL. The circle of Horii described by O'Driscoll et al stated that TTE could occur regardless of the status of aMCL (stage 3a). The authors speculated that the RH and coronoid fractures dissipated the energy of impact and stopped the progression of the circle before the elbow dislocation. Therefore, they supported that the stability of the elbow could be restored with the stabilization of the radio-capitellar joint (ie, LCL, RH, and CP repair). In 1998, Ring and Jupiter^26^ used a 4-column linkage theory (anterior, posterior, lateral, and medial columns) that has been compared to a ring to illustrate the restraints of the elbow. The stability of the elbow is guaranteed by the integrity of the different elements of the ring. Similarly, to pelvic ring injuries, stability is restored if both injured columns are repaired.

Despite this growing biomechanical data and related theories, a high incidence of TTE with MCL injury was reported in clinical studies.^27^ In 2012, a new unifying theory proposed a new circle of sequential injury starting from the medial side corresponding to a “reverse Horii circle”. Rhyou et al^26^ speculated that axial compression and valgus stress were the primary loads while hypersupination of the forearm was a secondary load. The primary load could cause the rupture of the MCL, which could induce—in combination with a hypersupination moment—a lateral translation strain on the ulna under the trochlea until the PL dislocation of the elbow, with potentially associated fractures of the RH and CP. The authors speculated that the LCUL was stripped when the RH was abutted against the posterior aspect of the capitulum. So the initial lesions started medially and ended laterally.

In 2018, Luokkala et al^17^ added a second circle to the reverse Horrii circle, equivalent to a spiral around the elbow. The authors considered the tendons less stiff than the ligaments and speculated that the medial and then lateral ligament complex would fail before the common flexor and then extensor mass origins.

#### Surgery: protocols and disagreements

In 2005, Mc Kee et al^18^ published the first surgical protocol for TTE. They recommended to systematic repair of the CP, RH, and LCL injuries via a lateral approach. Based on the sequential injury according to the Horii circle of O'Driscoll, this protocol allowed to reduce the rate of persistent instability and led to satisfactory outcomes at short- to mid-term follow-ups.^24^ However, recent biomechanical studies have changed the indication of fixations for CP fractures in TTE.^8^

A new classification system for CP fractures published by described 3 types of fractures based on both their amount and anatomic location: tip (I), anteromedial (II), and base (III).^22^ In 2012 Adams et al added the mid-transverse (50% of the CP height), and anterolateral fractures of the CP. According to Doonberg et al,^3^ the tip and mid-transverse fractures of the coronoid (<50% of CP height) represented 97% of TTE. In 2012, Jeon et al^12^ showed that fixation of all CP fractures was not necessary if the fracture involved less than 50% of the coronoid, and if the LCL and RH were fixed or intact. In the same years, Hartzler et al^8^ confirmed the less impact of the fixation of mid-transverse fracture on valgus and external rotation laxity. However, the authors showed significant increase in the stability in varus and internal rotation after CP fixation, regardless the status of the RH. Therefore, the authors recommended fixing the CP fractures according to the varus stress test and/or the height and location of the CP fractures on the computed tomography scan.

However, the prevalence of PTOA is still elevated at mid- (11.2% at 3 years) and long-term follow-ups (66% at 9 years) regardless of the surgical treatments of TTE.^9^ The high rate of PTOA may be due to the initial cartilage lesions following the trauma.^9^ However, for Jung et al,^16^ the rate of PTOA after TTE was significantly higher if the MCL was not repaired. Eygendaal et al^5^ showed an increased medial space opening under valgus stress in the elbow with a rupture of the MCL positioned at 90° of flexion. This instability cannot be detected by the usual stability tests of the elbow and could be equivalent to subclinical instability or micro-instability.^30^ In 2010, Jeong et al^13^ showed that the MCL repair in TTE prevented the onset of moderate or severe PTOA in 13 patients. However, the literature does not provide any data about the long-term radiographic outcomes of the TTE according to the MCL status. Further studies are necessary to assess the risk factor of PTOA after TTE in the long-term.

## Lesson learned

Since McKee’s protocol, the consensus is to first stabilize the radio-capitular joint by addressing the RH and LCUL injuries. The fixation of the coronoid fracture will depend on the residual stability after the lateral complex fixation.

**Figure 2:**
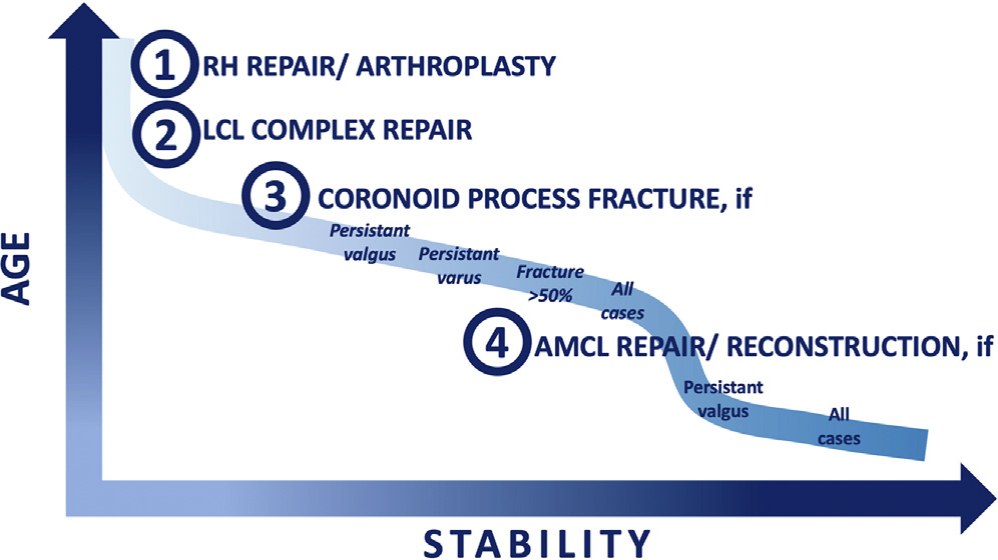
The proposition of treatment algorithm for terrible triad injuries of the elbow. *RH*, radial head; *LCL*, lateral collateral ligament.

The formalization of the TTE treatment prevents gross instability and avoids terrible short- to mid-term outcomes. However, PTOA at long-term follow-up is still an issue despite the improvement of our biomechanical knowledge and surgical protocols. Recent data suggest that a deficient aMCL and CP fracture (ie, mid-transverse and tip) could induce an infra clinical valgus or varus instability. Thus, it seems that the aMCL and the coronoid should be addressed to prevent long-term complication rates in younger or athlete patient population (Fig. 2).

## Conclusions

In conclusion, there exists no consensus on the surgical protocol for the treatment of TTE has been provided in the literature. While the outcomes of the TTE have been improved, the rate of PTOA at long-term follow-up is still high regardless of the treatments. The terrible triad has given way to the subtle triad with persistent microinstability of the elbow. Based on this historical review, we argue that the next challenge for elbow surgeons is to diagnose and fix this persistent subclinical instability after surgery for TTE in order to prevent the onset of PTOA.

## Disclaimers

Funding: No funding was disclosed by the authors.

Conflicts of interest: The authors, their immediate families, and any research foundation with which they are affiliated have not received any financial payments or other benefits from any commercial entity related to the subject of this article.

## References

[1] Carr C.R., Howard J.W. (1951). Metallic cap replacement of radial head following fracture. West J Surg Obstet Gynecol.

[2] Cutler C.W. (1926). Fractures of the head and neck of the radius. Ann Surg.

[3] Doornberg J.N., Ring D. (2006). Coronoid fracture patterns. J Hand Surg.

[4] Essex-Lopresti P. (1951). Fractures of the radial head with distal radio-ulnar dislocation; report of two cases. J Bone Joint Surg Br.

[5] Eygendaal D., Olsen B.S., Jensen S.L., Seki A., Söjbjerg J.O. (1999). Kinematics of partial and total ruptures of the medial collateral ligament of the elbow. J Shoulder Elbow Surg.

[6] Gutierrez L.S. (1964). A contribution to the study of the limiting factors of elbow extension. Cells Tissues Organs.

[7] Hassmann G.C., Brunn F., Neer C.S. (1975). Recurrent dislocation of the elbow. J Bone Joint Surg Am.

[8] Hartzler R.U., Llusa-Perez M., Steinmann S.P., Morrey B.F., Sanchez-Sotelo J. (2014). Transverse coronoid fracture: when does it have to be fixed?. Clin Orthop Relat Res.

[9] Heijink A., Vanhees M., van den Ende K., van den Bekerom M.P., van Riet R.P., Van Dijk C.N. (2016). Biomechanical considerations in the pathogenesis of osteoarthritis of the elbow. Knee Surg Sports Traumatol Arthrosc.

[10] Hotchkiss R.N. (1996).

[11] Hotchkiss R.N., Weiland A.J. (1987). Valgus stability of the elbow. J Orthop Res.

[12] Jeon I.H., Sanchez-Sotelo J., Zhao K., An K.N., Morrey B.M. (2012). The contribution of the coronoid and radial head to the stability of the elbow. J Bone Joint Surg Br.

[13] Jeong W.K., Oh J.K., Hwang J.H., Hwang S.M., Lee W.S. (2010). Results of terrible triads in the elbow: the advantage of primary restoration of medial structure. J Orthop Sci.

[14] Josefsson P.O., Gentz C.F., Johnell O., Wendeberg B. (1989). Dislocations of the elbow and intraarticular fractures. Clin Orthop Relat Res.

[15] Josefsson P.O., Johnell O., Wendeberg B. (1987). Ligamentous injuries in dislocations of the elbow joint. Clin Orthop Relat Res.

[16] Jung S.W., Kim D.H., Kang S.H., Eho Y.J., Yang S.W., Lee G.E. (2019). Risk factors that influence subsequent recurrent instability in terrible triad injury of the elbow. J Orthop Trauma.

[17] Luokkala T., Temperley D., Basu S., Karjalainen T.V., Watts A.C. (2019). Analysis of magnetic resonance imaging–confirmed soft tissue injury pattern in simple elbow dislocations. J Shoulder Elbow Surg.

[18] McKee M.D., Pugh D.M., Wild L.M., Schemitsch E.H., King G.J. (2005). Standard surgical protocol to treat elbow dislocations with radial head and coronoid fractures. Surgical technique. J Bone Joint Surg Am.

[19] Morrey B.F., An K.-N. (1983). Articular and ligamentous contributions to the stability of the elbow joint. Am J Sports Med.

[20] Morrey B.F., Tanaka S., An K.N. (1991). Valgus stability of the elbow. A definition of primary and secondary constraints. Clin Orthop Relat Res.

[21] O'Driscoll S.W., Morrey B.F., Korinek S., An K.N. (1992). Elbow subluxation and dislocation. A spectrum of instability. Clin Orthop Relat Res.

[22] O'Driscoll S.W., Jupiter J.B., Cohen M.S., Ring D., McKee M.D. (2003). Difficult elbow fractures: pearls and pitfalls. Instr Course Lect.

[23] Osborne G., Cotterill P. (1966). Recurrent dislocation of the elbow. J Bone Joint Surg Br.

[24] Pugh D.M., Wild L.M., Schemitsch E.H., King G.J., McKee M.D. (2004). Standard surgical protocol to treat elbow dislocations with radial head and coronoid fractures. J Bone Joint Surg Am.

[25] Regan W., Morrey B. (1989). Fractures of the coronoid process of the ulna. J Bone Joint Surg Am.

[26] Rhyou I.H., Kim Y.S. (2012). New mechanism of the posterior elbow dislocation. Knee Surg Sports Traumatol Arthrosc.

[27] Schreiber J.J., Warren R.F., Hotchkiss R.N., Daluiski A. (2013). An online video investigation into the mechanism of elbow dislocation. J Hand Surg.

[28] Speed K. (1941). Ferrule caps for the head of the radius. Surg Gynecol Obstet.

[29] Tullos H.S., Schwab G., Bennett J.B., Woods G.W. (1981). Factors influencing elbow instability. Instr Course Lect.

[30] Zwerus E.L., Somford M.P., Maissan F. (2018). Physical examination of the elbow, what is the evidence? A systematic literature review. Br J Sports Med.

